# Highlighting the Prognostic Importance of Measurable Residual Disease Among Acute Myeloid Leukemia Risk Factors

**DOI:** 10.4274/tjh.galenos.2020.2020.0157

**Published:** 2021-06-01

**Authors:** Zehra Narlı Özdemir, Uğur Şahin, Klara Dalva, Mehmet Akif Baltacı, Atilla Uslu, Cemaleddin Öztürk, Güldane Cengiz Seval, Selami Koçak Toprak, Meltem Kurt Yüksel, Pervin Topçuoğlu, Önder Arslan, Muhit Özcan, Meral Beksaç, Osman İlhan, Günhan Gürman, Sinem Civriz Bozdağ

**Affiliations:** 1 Ankara City Hospital, Clinic of Hematology, Ankara, Turkey; 2 Medicana International Ankara Hospital, Clinic of Hematology, Ankara, Turkey; 3 Ankara University Faculty of Medicine, Department of Hematology, Ankara, Turkey; 4 Ankara University Faculty of Medicine, Department of Internal Medicine, Ankara, Turkey

**Keywords:** Acute myeloid leukemia, Measurable residual disease, Multiparameter flow cytometry

## Abstract

**Objective:**

The optimal timing of measurable residual disease (MRD) evaluation in acute myeloid leukemia (AML) patients has not been well defined yet. We aimed to investigate the impact of MRD in pre- and post-allogeneic hematopoietic stem cell transplantation (AHSCT) periods on prognostic parameters.

**Materials and Methods:**

Seventy-seven AML patients who underwent AHSCT in complete morphological remission were included. MRD analyses were performed by 10-color MFC and 10^-4^ was defined as positive. Relapse risk and survival outcomes were assessed based on pre- and post-AHSCT MRD positivity.

**Results:**

The median age of the patients was 46 (range: 18-71) years, and 41 (53.2%) were male while 36 (46.8%) were female. The median follow-up after AHSCT was 12.2 months (range: 0.2-73.0). The 2-year overall survival (OS) in the entire cohort was 37.0%, with a significant difference between patients who were MRD-negative and MRD-positive before AHSCT, estimated as 63.0% versus 16.0%, respectively (p=0.005). MRD positivity at +28 days after AHSCT was also associated with significantly inferior 2-year OS when compared to MRD negativity (p=0.03). The risk of relapse at 1 year was 2.4 times higher (95% confidence interval: 1.1-5.6; p=0.04) in the pre-AHSCT MRD-positive group when compared to the MRD-negative group regardless of other transplant-related factors, including pre-AHSCT disease status (i.e., complete remission 1 and 2). Event-free survival (EFS) was significantly shorter in patients who were pre-AHSCT MRD-positive (p=0.016). Post-AHSCT MRD positivity was also related to an increased relapse risk. OS and EFS were significantly inferior among MRD-positive patients at +28 days after AHSCT (p=0.03 and p=0.019).

**Conclusion:**

Our results indicate the importance of MRD before and after AHSCT independently of other factors.

## Abstract (tr)

**Amaç:**

Akut myeloid lösemi (AML) hastalarında ölçülebilir kalıntı hastalık (MRD) değerlendirmesinin optimal zamanlaması henüz tam olarak tanımlanmamıştır. Allojeneik hematopoietik kök hücre nakli (AHKHN) öncesinde ve sonrasında MRD’nin prognostik parametreler üzerindeki etkisini araştırdık.

**Gereç ve Yöntemler:**

Tam morfolojik remisyonda AHKHN yapılan 77 AML hastası çalışmaya dahil edildi. MRD analizleri 10 renkli akım sitometri ile yapıldı ve 10^-4^ pozitif olarak tanımlandı. Nüks riski ve sağkalım sonuçları AHKHN öncesi ve sonrası MRD pozitifliğine göre değerlendirildi.

**Bulgular:**

Hastaların ortanca yaşı 46 (18-71) yıl olup, bunların 41’i (%53,2) erkek, 36’sı (%46,8) kadındı. AHKHN sonrası medyan takip süresi 12,2 aydı (0,2-73 ay). Tüm kohortta 2 yıllık genel sağkalım (OS) %37 olup, nakil öncesi MRD-negatif ve MRD-pozitif olan hastalar arasında anlamlı sağkalım farkı saptandı (%63 vs %16, p=0,005). İki yıllık OS AHKHN sonrası +28 günde MRD pozitif olan hastalarda, MRD negatif olanlar ile karşılaştırıldığında önemli ölçüde kısa bulundu (p=0,03). Bir yıllık nüks riski nakil öncesi hastalık durumu da dahil olmak üzere diğer nakil ile ilişkili faktörlerden bağımsız olarak, nakil öncesi MRD pozitif olanlarda MRD negatif hastalara kıyasla 2,4 kat (%95 güven aralığı: 1,1-5,6; p=0,04) daha yüksek bulundu. AHKHN öncesi MRD pozitif olan hastalarda olaysız sağkalım (EFS) anlamlı olarak daha kısaydı (p=0,016) ve nakil sonrası MRD pozitifliği artmış nüks riskiyle ilişkiliydi. OS ve EFS, nakil sonrası +28 günde MRD pozitif olan hastalar arasında daha kısa saptandı (p=0,03 ve p=0,019).

**Sonuç:**

Sonuçlarımız diğer faktörlerden bağımsız olarak AHKHN öncesi ve sonrası MRD’nin önemini göstermektedir.

## Introduction

Acute myeloid leukemia (AML) is a biologically aggressive and heterogeneous disease characterized by a large number of molecular abnormalities [1]. Although achievement of morphological complete remission (CR) is still an important end point, this cut-off allows for the presence of up to 10^10^ leukemic blasts or more [2]. Approximately two-thirds of CR patients may relapse within a few years after frontline therapy [3,4]. At diagnosis multiple factors have prognostic impact for outcome, including clinical parameters and cytogenetics, as well as molecular factors and biological properties of the leukemic cells. Risk factors at diagnosis were shown to correlate with quality of remission as reflected by measurable residual disease (MRD) [5,6].

Allogeneic hematopoietic stem cell transplantation (AHSCT) may be the curative treatment option for patients with AML. The outcome of AHSCT depends on various factors including conditioning regimen, CR status, cytogenetic risk group and molecular markers, graft-versus-host disease (GvHD) prophylaxis, and presence of chronic GvHD [7]. According to a report of the European Society for Blood and Marrow Transplantation, approximately 40% of AML patients will relapse after AHSCT and have poor prognosis with 2-year survival of <20% [7]. Transplant-related mortality and disease relapse remain the most significant barriers for long-term survival of AML patients.

In previous studies, MRD positivity after induction and post-remission therapy have been widely analyzed. However, the optimal timing of MRD monitorization in AML patients has not been clearly defined yet. Data about the impact of MRD in the AHSCT setting are limited [8,9].

In the present study, we analyzed AML patients undergoing AHSCT in morphological CR for whom pre- and post-AHSCT MRD assessments by multicolor flow cytometry were available. Besides ascertaining the relationship between pre/post-AHSCT MRD and post-AHSCT outcomes, we also investigated whether MRD is more important than other risk factors including conditioning regimen [myeloablative (MA) or non-MA (NMA)], pre-AHSCT disease status (CR_1_ or >CR_1_), cytogenetic risk, donor type, the presence of acute GvHD, and cytomegalovirus (CMV) reactivation.

## Materials and Methods

### Patients

We retrospectively evaluated 77 AML patients who were older than 18 years and underwent AHSCT while in morphological CR between January 2013 and December 2018 in the Ankara University School of Medicine. The patients without MRD data before and after AHSCT were excluded from the study.

The medical records of the Ankara University Faculty of Medicine were reviewed in terms of age, sex, conditioning regimen (MA or NMA), pre-AHSCT disease status (CR_1_ or >CR_1_), donor type [fully matched, single antigen-mismatched (SAM), or haploidentical], cytogenetic risk (favorable, standard, or high), presence of acute GvHD, CMV reactivation, and presence of MRD before AHSCT and at day +28 after AHSCT. Information on post-transplant outcomes was obtained via the follow-up program through medical records from our outpatient clinic.

The diagnosis of AML was based on clinical, morphological, and immunophenotypical features identified based on the 2008 revision of the World Health Organization (WHO) classification of AML and related neoplasms for those who were diagnosed before 2016 [10]. Revised WHO criteria were used to define AML after 2016 [11]. CR was defined as <5% blasts by morphology in pre-transplant BM aspirates. The 2017 European Leukemia Network risk stratification by genetics was used to assign cytogenetic risk [12].

### Detection of MRD

Multiparameter flow cytometry (MFC) was performed for all patients as a routine clinical test on bone marrow aspirates as a baseline assessment before AHSCT as well as on day +28 after AHSCT. MRD assessments were performed during pre-transplant workup and at days 28±7 after AHSCT in patients who achieved engraftment. Neutrophil and platelet engraftment were obtained in all patients before MRD assessment, except one who underwent a second AHSCT with haploidentical graft. Engraftment was defined as an absolute neutrophil count greater than 500 cells per liter (absolute neutrophil count >0.5x10^9^/L) on the first day of three consecutive days and platelet count greater than 20,000 cells per liter (platelet count >20x10^9^/L) on the first day of seven consecutive days without transfusion support.

Ten-color MFC was used for MRD assessment and MRD was identified by visual inspection as a cell population showing deviation. The approaches used to detect MRD by MFC were identification of leukemia-associated immunophenotypes that differed from the majority of normal hematopoietic cells and identification of different-from-normal patterns [13,14].

Core markers were selected for the backbone of the panel to identify myeloid blast populations, combined with markers from lymphoid/myelomonocytic maturation groups to define the AML MRD panel. An AML MRD panel consisting of antibody combinations recognizing CD4, CD5, CD7, CD11b, CD13, CD14, CD15, CD16, CD19, CD33, CD34, CD38, CD45, CD45RA, CD56, CD64, CD71, CD117, CD123, and HLA-DR was used for MRD detection. A total of 100,000 to 500,000 nucleated cells were examined and 10^-4^ was the threshold for the sensitivity of MRD detection [15]. The acquisition of the cells was performed using a Navios flow cytometer (3-laser, 10-color, Beckman Coulter). The collected data were analyzed using Kaluza software (Navios, Beckman Coulter, USA). When an abnormal population was identified, it was quantified as a percentage of the total CD45+ white cell events. Any measurable level of MRD was considered positive [16].

### Statistical Analysis

The main objective of this study was detecting a significant survival advantage, if any, among MRD-negative patients (either pre- or post-AHSCT) when compared to MRD-positive patients. Relapse and transplant-related mortality rates were also evaluated as secondary objectives. Three major outcomes were assessed accordingly: overall survival (OS), event-free survival (EFS), and non-relapse mortality (NRM), which were calculated from the time of transplant. The survival estimations were performed by Kaplan-Meier method and the log-rank test was used for comparison of survival distribution among groups. Patient age, sex, donor type, cytogenetic risk, disease status at AHSCT (CR_1_ or >CR_1_), type of conditioning regimen, presence of CMV reactivation, and cumulative incidences of acute and chronic GvHD were compared by chi-square, Fisher exact, Student t, and Mann-Whitney U tests as appropriate. GvHD could not be calculated as a cumulative incidence function or considered a competing mortality risk due to a lack of data on GvHD onsets.

Retrospective power analysis with a two-sided log rank test demonstrated 86.0% power at a 0.05 significance level to detect a difference of 0.25 between 0.63 and 0.38, which are the calculated 1-year estimated OS rates in the pre-AHSCT MRD-negative and MRD-positive cohorts, respectively.

Cox regression analysis was used to determine the effects of MRD positivity adjusted for potential confounding factors. In order to analyze the effects of factors associated with at least borderline significance (p<0.20) in the univariate analysis on the outcomes of OS, EFS, and NRM, they were entered via backwards selection into a Cox proportional hazards model, which was assessed by means of residual (Schoenfeld and Martingale) analysis. Cohort size limited the number of factors in each model to those with suggested association in univariate analysis. Multivariate analyses were performed for only pre-AHSCT MRD since no factors with statistical significance were suggested in the univariate analyses of post-AHSCT MRD. Multivariate analyses for pre-AHSCT MRD included pre-AHSCT disease status and the presence of acute GvHD as potential confounders.

The statistical software packages PASS version 11.0 (NCSS, LLC, Kaysville, UT, USA) and IBM SPSS Statistics for Windows version 25.0 (IBM Corp., Armonk, NY, USA) were used for power analysis and for the rest of the statistical analysis, respectively. Type I error of 5% (two-sided) was used to infer statistical significance in all analyses.

## Results

Forty-one male (53.2%) and 36 female (46.8%) patients were included in the study (n=77). The median age was 46 (range: 18-71) years. Nine (11.7%) patients had favorable cytogenetic risk profiles and 68 (88.3%) patients had standard-high risk. t(v;11q23.3) (MLL rearranged) was observed in 1 patient, inversion 16 (inv 16) in 3 patients, t(8,21) in 3 patients, and *NPM1* mutation in 3 patients at the pre-transplantation workup. Forty-four (57.1%) patients underwent AHSCT from a fully matched donor, 27 (35.1%) from a SAM donor, and 6 (7.8%) from a haploidentical donor. Fifty-two (67.5%) and 25 (32.5%) patients underwent transplantation in CR_1_ and CR_2_, respectively. Sixty-four (83.1%) patients received MA and 13 (16.9%) NMA conditioning regimens. Forty-four (57.1%) patients were pre-AHSCT MRD-negative whereas 43 (55.8%) of patients had MRD negativity at post-transplantation assessment (Table 1). Of the 44 patients who were MRD-negative prior to transplant, 34 (77%) remained MRD-negative and 10 patients (22.7%) had detectable MRD. Of the 33 patients MRD-positive before transplant, 9 achieved MRD negativity after transplantation (Table 2).

### Relationships Among MRD Status, Survival, Relapse, and NRM

No significant differences were observed between MRD-negative and MRD-positive groups in terms of age, cytogenetic risk, donor type, pre-AHSCT disease status (CR_1_ or >CR_1_), conditioning regimen, presence of acute and chronic GvHD, or CMV reactivation, neither in the pre-AHSCT nor the post-AHSCT period (p>0.05) (Table 3).

**Figure 1:**
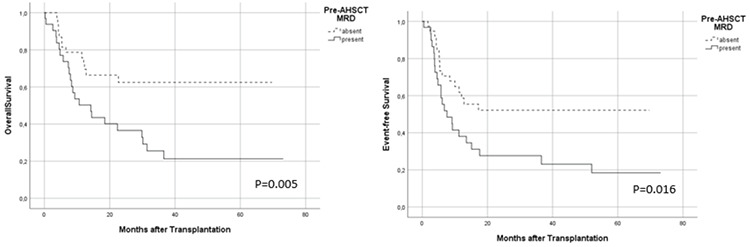
Association between pre-AHSCT MRD status and transplant outcomes. AHSCT: Allogeneic hematopoietic stem cell transplantation; MRD: measurable residual disease.

**Figure 2:**
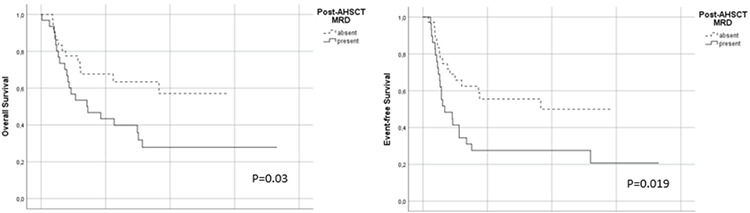
Association between post-AHSCT MRD status and transplant outcomes. AHSCT: Allogeneic hematopoietic stem cell transplantation; MRD: measurable residual disease.

The median follow-up after AHSCT was 12.2 months (range: 0.2-73.0) with no patients lost to follow-up. The risk of relapse at 1 year was estimated to increase by 2.4 times [95% confidence interval (CI): 1.1-5.6; p=0.04] in the pre-AHSCT MRD-positive group when compared to the MRD-negative group. EFS was significantly shorter in patients who had pre-AHSCT MRD positivity (p=0.016; Figure 1). Post-AHSCT MRD positivity was also associated with increased relapse risk. EFS was significantly poorer in patients who were MRD-positive on day +28 after AHSCT (p=0.019) (Figure 2). MRD positivity before and after AHSCT did not show a significant association with NRM (p=0.97 and 0.56, respectively).

The 2-year estimate of OS in the entire cohort was 37.0%. A significant difference in OS was observed between patients who were MRD-negative and MRD-positive before AHSCT, estimated as 63.0% versus 16.0% at 2 years (p=0.005) (Figure 1). Patients who were MRD-negative on day +28 after AHSCT had higher OS rates when compared to MRD-positive patients at 1 year (63.0% vs. 41.0%) and at 2 years (55.0% vs. 23.0%), respectively (p=0.03) (Figure 2).

**Figure 3:**
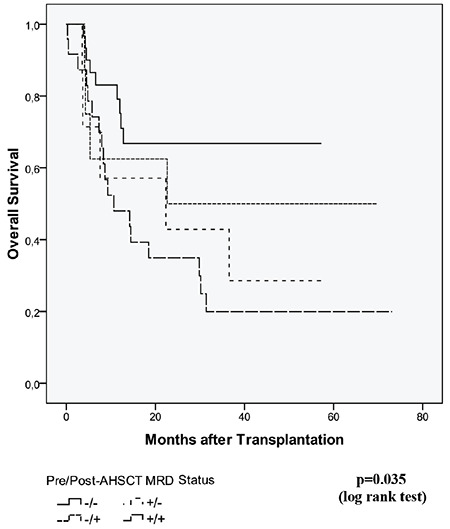
Overall survival according to pre- and post-AHSCT MRD status. AHSCT: Allogeneic hematopoietic stem cell transplantation; MRD: measurable residual disease.

**Figure 4:**
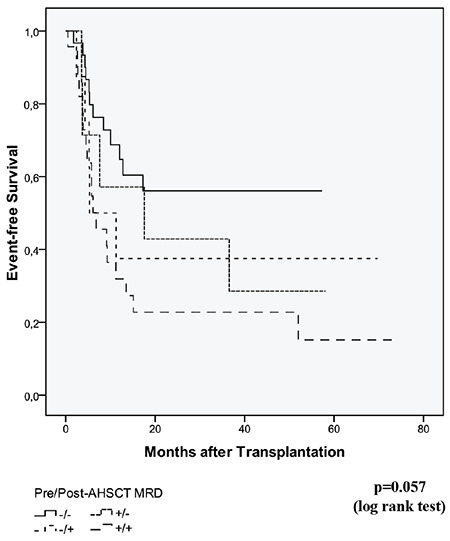
Event-free survival according to pre- and post-AHSCT MRD status. AHSCT: Allogeneic hematopoietic stem cell transplantation; MRD: measurable residual disease.

Patients who were MRD-negative before and after AHSCT had the best OS and EFS (p=0.035 and 0.057, respectively). Patients who underwent AHSCT with positive MRD status and those who came out of the transplant again with the presence of MRD had the worst OS and EFS (p=0.035 and 0.057, respectively) (Figures 3 and 4). In Cox regression analysis, patients with negative MRD status before and after AHSCT had significantly better OS and EFS compared with patients who were MRD-positive before and after transplantation (p=0.006 and 0.008, respectively). OS and EFS were better in patients with pre-AHSCT MRD negativity and post-AHSCT MRD positivity and those with pre-AHSCT MRD positivity and post-AHSCT MRD negativity compared with patients with pre- and post-AHSCT positive MRD status; however, this did not reach statistical significance.

**Figure 5:**
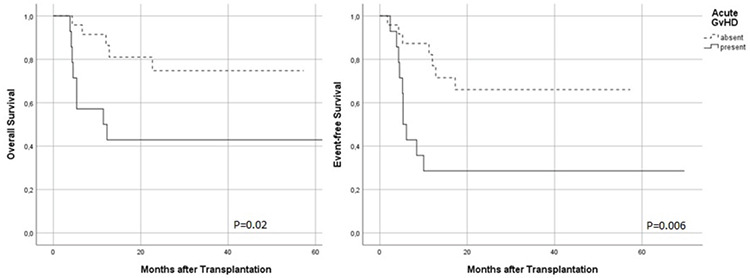
The impact of acute GvHD on survival among pre-AHSCT MRD-negative patients. AHSCT: Allogeneic hematopoietic stem cell transplantation; MRD: measurable residual disease; GvHD: graft-versus-host disease.

In the pre-AHSCT MRD-negative group, the presence of acute GvHD was related to inferior OS and EFS rates (p=0.02 and 0.006, respectively) (Figure 5). Acute GvHD occurred in 27 (35%) patients, cutaneous acute GvHD in 14 (51.9%) patients, gastrointestinal system (GIS) GvHD in 7 (25.9%) patients, cutaneous and GIS acute GvHD in 3 (11.1%) patients, GIS and liver acute GvHD in 2 (7.4%) patients, and cutaneous, GIS, and liver acute GvHD in 1 (3.7%) patient. Grade 3-4 acute GvHD according to the Glucksberg criteria was observed in 10 (37%) patients. Multivariate analyses including pre-AHSCT MRD status and the presence of acute GvHD showed that an OS advantage remained in patients who were MRD-negative in the pre-AHSCT period even if acute GvHD occurred after transplantation (hazard ratio: 2.5, 95% CI: 1.3-4.9; p=0.008).

In the presence of pre-AHSCT MRD, other variables known to have prognostic significance including age, cytogenetic risk, donor type, pre-AHSCT disease status (CR_1_ or >CR_1_), and conditioning regimen had no effect on the transplant outcome. The presence of these variables was not strong enough to change the negative effect of the presence of pre-AHSCT MRD.

## Discussion

The presence of MRD is a strong, independent prognostic marker of increased risk of relapse and shorter survival in patients with AML. Testing for MRD can be used to refine risk stratification and treatment response assessment, and it may help guide post-remission treatment strategies like proceeding with AHSCT or not [17]. The optimal timing of MRD assessment has not been exactly defined. However, MRD after induction and remission has been studied. The HOVON Group prospectively evaluated bone marrow specimens of 389 patients younger than 61 years. After all courses of therapy, low MRD values distinguished patients with relatively favorable outcomes from those with high relapse rates and adverse relapse-free survival (RFS) and OS. They showed that residual disease detected by MFC was related to higher 4-year relapse risk (72% and 42%, respectively) and adverse RFS at 4 years (23% and 52%, respectively) [18]. Also, MRD analysis in the pre- and post-transplantation settings may have a crucial role in long-term outcomes. In our study, we identified 33 patients who had MRD positivity before transplantation, and 9 of these patients could achieve MRD negativity in the post-transplantation period. The presence of pre-AHSCT MRD was related to a significantly higher (2.4-fold) relapse rate and shorter EFS. There was no difference in NRM rates between the pre-AHSCT MRD-positive and MRD-negative groups. We also found OS to be significantly lower in pre-transplant MRD-positive patients. Our results were comparable with those of previous studies. Oran et al. [19] showed that MRD status at transplantation could independently predict 1-year relapse incidence in patients with AML. Relapse incidence at 1 year was higher in AML patients with MRD (32.6% vs. 14.4%, p=0.002). Leukemia-free survival (43.6% vs. 64%, p=0.007) and OS (48.8% vs. 66.9%, p=0.008) rates were also inferior in patients with MRD [19]. A meta-analysis reported that pre-transplant MRD was associated with worse leukemia-free survival, OS, and cumulative incidence of relapse but not NRM. Associations between MRD status and outcome held regardless of MRD detection method, intensity of conditioning regimen, and patient age [2]. In our study, in cases of pre-AHSCT MRD, conditioning regimen intensity did not show any impact on outcome. All MRD detection was performed by flow cytometry so we did not analyze the influence of the detection method.

Walter et al. suggested that pre-AHSCT MRD by MFC is associated with increased risk of relapse and death after MA AHSCT for AML patients in CR_1_ regardless of other risk factors. Two-year estimates of OS were 30.2% and 76.6% for MRD-positive and MRD-negative patients while 2-year estimates of relapse were 64.9% and 17.6%, respectively [13]. In a subsequent study, they reported similar outcomes in patients who underwent AHSCT in CR_1_ or CR_2_, which was significantly dependent on MRD status prior to transplant [20]. Similarly to that study, we showed MRD positivity as an independent factor regardless of performing transplantation in CR_1_ or CR_2_.

Relapse after AHSCT remains a problem in AML patients. Can MRD follow-up after AHSCT predict relapse and improve the outcomes? In one study, MRD positivity at the 30^th^ post-transplantation day predicted the relapse risk in 1 year (group 1: 1-year relapse incidence, 78%) [21]. The authors claimed that the positivity of MRD at any time after transplantation in patients with morphological CR was related to relapse that might occur within 2 months. In our study, 34 patients were MRD-positive at the 28^th^ day after transplantation, and 24 of them were those in the pre-AHSCT group. We showed that MRD positivity on the 28^th^ post-transplantation day was related to significantly higher relapse risk and poor EFS and OS in patients with AML.

However, there is a relative lack of data regarding MRD and MRD-guided interventions following AHSCT. Post-transplant MRD is related to an increased incidence of relapse, but clinical effects of MRD kinetics are not clearly defined yet [22]. Platzbecker et al. [23] reported that MRD-guided treatment with azacitidine can prevent or delay hematological relapse in patients with myelodysplastic syndrome and AML. Discontinuation of immunosuppression and donor lymphocyte infusion (DLI) may be beneficial in patients with post-AHSCT MRD, but there is no convincing evidence that preventive intervention strategies will improve the outcome [22]. Our institutional policy is to taper immunosuppression or intervene with DLI. Targeted therapies like Flt3 inhibitors have also been preferred in patients with mutations. However, a limitation of our study is our not analyzing the impact of these therapies on outcome per patient.

## Conclusion

Our study showed that the presence of MRD both in pre- and post-transplantation settings was related to significantly poorer outcomes as an independent prognostic marker for increased relapse risk and shorter survival for AML patients.

## Figures and Tables

**Table 1:**
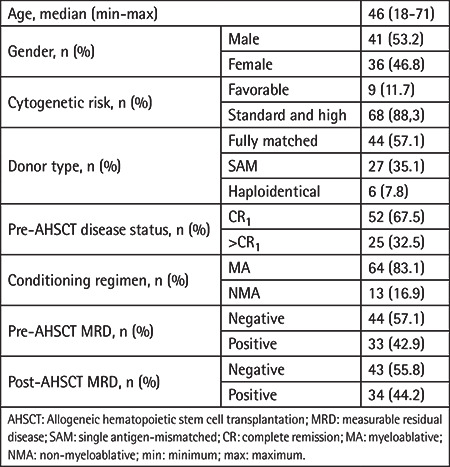
General characteristics of patients.

**Table 2:**
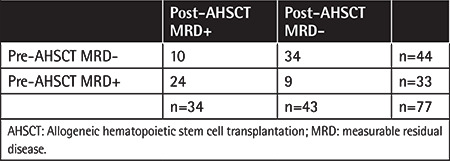
Changes of MRD status according to AHSCT.

**Table 3:**
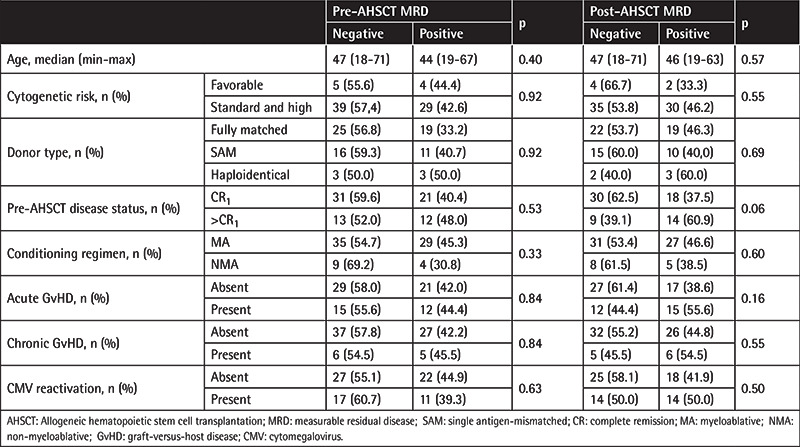
Distribution of studied parameters according to pre- and post-MRD positivity.
